# phenosim - A software to simulate phenotypes for testing in genome-wide association studies

**DOI:** 10.1186/1471-2105-12-265

**Published:** 2011-06-29

**Authors:** Torsten Günther, Inka Gawenda, Karl J Schmid

**Affiliations:** 1 Institute of Plant Breeding, Seed Science and Population Genetics, University of Hohenheim, Stuttgart, Germany

## Abstract

**Background:**

There is a great interest in understanding the genetic architecture of complex traits in natural populations. Genome-wide association studies (GWAS) are becoming routine in human, animal and plant genetics to understand the connection between naturally occurring genotypic and phenotypic variation. Coalescent simulations are commonly used in population genetics to simulate genotypes under different parameters and demographic models.

**Results:**

Here, we present phenosim, a software to add a phenotype to genotypes generated in time-efficient coalescent simulations. Both qualitative and quantitative phenotypes can be generated and it is possible to partition phenotypic variation between additive effects and epistatic interactions between causal variants. The output formats of phenosim are directly usable as input for different GWAS tools. The applicability of phenosim is shown by simulating a genome-wide association study in *Arabidopsis thaliana*.

**Conclusions:**

By using the coalescent approach to generate genotypes and phenosim to add phenotypes, the data sets can be used to assess the influence of various factors such as demography, genetic architecture or selection on the statistical power of association methods to detect causal genetic variants under a wide variety of population genetic scenarios. phenosim is freely available from the authors' website http://evoplant.uni-hohenheim.de

## Background

In recent years, genome-wide association studies (GWAS) became widely used to uncover the genetic basis of complex traits by comparing patterns of genetic and phenotypic variation [1-3]. The power of such studies depends on various factors that include the genetic architecture of the trait, the demographic history of the population, and variation in mutation and recombination rates [4]. In addition, the trait under investigation may be adaptive or (in case of a disease trait) can evolve under purifying selection, which both would result in a non-neutral pattern of genetic diversity in the genomic neighborhood of the causal mutation.

Coalescent simulations are widely used to simulate genotypes under complex demographies [5] with recent extensions to include recombination hotspots [6] and selection [7], or to simulate whole genomes [8]. Simulations are often used to test population genetic hypotheses by comparing simulated and observed data. However, such simulations produce only genotypes but not phenotypes, which are also required to test methods for detecting significant associations between genetic and phenotypic variation. Although some tools provide an option to map phenotypes onto simulated genotypes, they only allow the simulation of qualitative phenotypes [9] or require time-consuming forward-in-time simulations to create genotypes from complex demographic scenarios [10-13].

Here, we present phenosim, a tool written in Python [14] that was designed to add a phenotype to genotypes simulated by coalescent-based simulation tools. Simulated phenotypes may either be qualitative or quantitative traits with different effect sizes and may show epistatic interactions. Hence, the simulation of case/control studies as well as the search for quantitative trait nucleotides (QTNs) of a complex trait with a user-defined architecture is possible. By combining simulated genotypes and phenotypes, researchers can assess the influence of different factors on the power of new methods for association mapping, compare different methods or estimate an optimal sample size and number of markers for a given study design.

## Implementation

**Figure 1:**
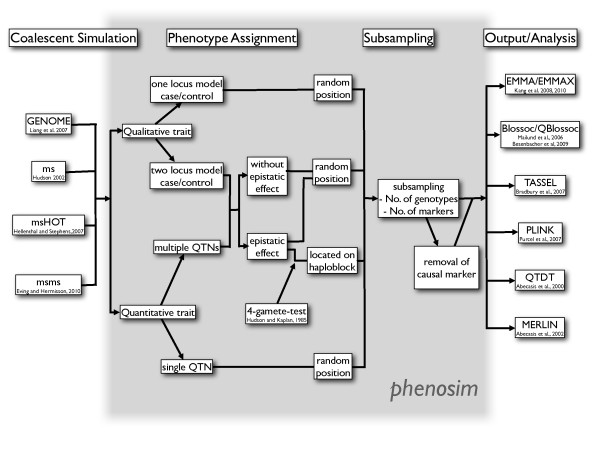
**Work flow**. Flowchart of the phenosim pipeline.

The general work flow of phenosim is shown in Figure 1. First, the user simulates genotypes with one of four different programs for coalescent simulations. In the current version, phenosim is able to read the output of the ms[5], msHOT[6], msms[7] and GENOME[8] programs. After the import of genotypes, a phenotype generated under a user-defined model is assigned to each genotype. The trait can either be qualitative or quantitative.

For qualitative traits, one- and two-locus models are supported. The user defines the model by setting the penetrance (probability of being affected) for all genotypes. In the two-locus model, this is done by a penetrance table for all possible allelic combinations among the two loci. Therefore, the user may define arbitrary interactions between all alleles of the loci. The case/control-status of all simulated individuals is then assigned according to the model. In many cases, disease states are caused by risk alleles segregating at low allele frequencies in the overall population. As such low frequency variants share a genealogy that may differ from high frequency variants and thus the linkage pattern around these variants may be different [15], the user can restrict causal mutations to a certain frequency range to obtain realistic risk loci. However, as this may result in a low number of cases in the final sample, users need to simulate larger populations and optionally enter a minimum number of cases to be sampled from the population. This procedure reflects the sampling procedure of many case/control studies.

For quantitative traits, multiple QTNs with additive effects or epistatic interactions between two QTNs are possible. By default locations of causal variants are selected randomly. Nevertheless, the user can determine the position of a QTN manually and/or restrict the selection to an allele frequency range. A phenotype is generated based on the formulas of [16], which we generalize for additive effects among multiple QTNs as follows. The trait value is calculated by adding a fixed variance proportion explained by the QTN to a random number drawn from a standard normal distribution with mean 0 and standard deviation 1, *N*(0, 1). We provide two different models, depending on the ploidy of the individuals. The effect of the *j*-th QTN is *π_j _*and the QTN has a derived allele frequency of *f_j_*. It should be noted that the sum of all QTN effects, ∑*_j _π_j_*, equals the heritability, *h*^2^, of the trait. If the individuals are haploid, the allelic state of the *i*-th individual at the *j*-th QTN is *a_ij_*, where *a_ij _*:= 0 if the allele is ancestral and *a_ij _*:= 1 if it is derived. Then the phenotype *Y *of individual *i *is calculated as:(1)

The phenotype of diploid individuals under an additive model without dominance is calculated as:(2)

where *Q_ij _*:= 1 if the *j*-th QTN is homozygous derived, *Q_ij _*:= 0 if the QTN is heterozygous and *Q_ij _*:= -1 if the QTN is homozygous ancestral. Dominant effects at each QTN and additive effects between loci are also supported for diploids. In this case, equation (1) is used with *a_ij _*:= 0 for homozygous ancestral QTNs and *a_ij _*:= 1 for heterozygous and homozygous derived individuals.

If exactly two QTNs are selected, a positive, additive epistatic effect *π_E _*between these QTNs can be simulated. This epistasis is modeled as a fictive third QTN, whose allelic state *a_iE _*is 1, if the individual carries at least one derived allele at both basal QTNs. For users with a some Python scripting experience, other types of epistasis can easily be simulated by modifying the code of phenosim. To simulate a causal haplotype or allelic heterogeneity among two causal variants within a single gene, both QTNs may also be located on a common haploblock defined by the four-gamete test [17].

To our knowledge, quantiNemo[12] is the only software that currently supports the simulation of interactions between QTNs. However, quantiNemo utilizes time-consuming forward simulations, whereas phenosim allows to include epistasis between QTNs within a time-efficient coalescent framework.

 After phenotypes have been generated, a predefined number of markers and/or individuals can be sub sampled from the total simulated population. The causal marker(s) can be optionally removed from the sample, since frequently the causal mutation itself is not genotyped in a genome-wide study. Finally, genotypes and phenotypes are written into different output file formats that can be directly used as input for commonly used association programs such as Blossoc/QBlossoc[16,18], EMMA/EMMAX[19,20], PLINK[21], QTDT/MERLIN[22,23] and TASSEL 3.0 [24]. A snapshot of phenosim is available as Additional File 1 whereas the most current version is maintained at http://evoplant.uni-hohenheim.de

## Results and Discussion

**Figure 2:**
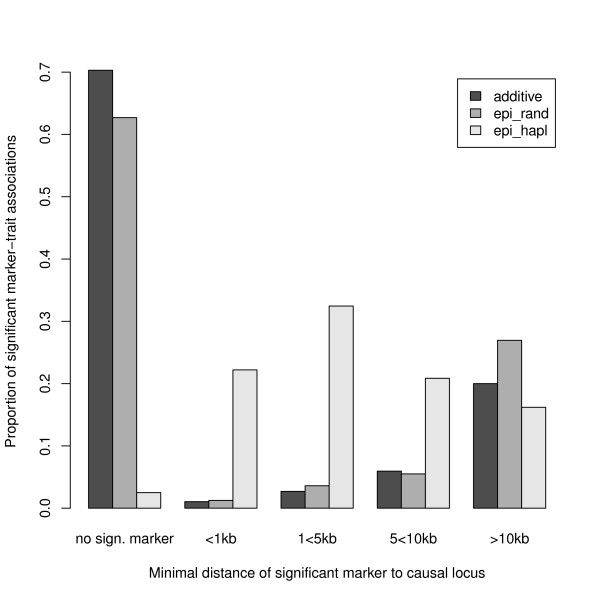
**Example**. Proportion of significant marker-trait associations (Bonferroni adjusted significance threshold) found at different distances from the causal marker. For each QTN, the distance to the next significant association is shown. The bars on the left show the proportion of simulated data sets for which no significant association was found. The simulated models include: 'additive' - 2 QTNs randomly distributed with a variance proportion of 0.05 each; 'epi_rand' - 2 QTNs randomly distributed with a variance proportion of 0.01 each and 0.08 epistatic effect; 'epi_hapl' - 2 QTNs located on a common haploblock with a variance proportion of 0.01 each and 0.08 epistatic effect. Each model was simulated 1000 times.

To demonstrate the ability of phenosim to simulate data for GWAS, we utilized GENOME[8] and simulated populations *N_e _*= 1000, with a population recombination parameter of *ρ *= 8 *· *10^-3 ^and 250,000 SNPs distributed over a 120 Mbp genome. These settings are comparable to data sets used for recent GWAS in *A. thaliana *[2,25,26]. phenosim was then used to generate phenotypes under three different models: (i) 2 QTNs, each with an effect of 0.05; (ii) 2 QTNs at random positions, each with an effect of 0.01, and epistatic interaction of *π_E _*= 0.08; and (iii) 2 QTNs, located on a common haploblock, each with an effect of 0.01 and epistatic interaction of *π_E _*= 0.08. In all three scenarios, the total proportion of variance explained by these QTNs and their interaction was identical (*h*^2 ^= 0.1). Four hundred chromosomes were sub-sampled and the causal polymorphisms were removed from the data. EMMAX[20] was used to detect marker-trait associations and the causal locus for this hypothetical trait. In Figure 2 we show the proportion of significant markers that were found at a given distance from the causal locus. In the first model (only additive effects), less than 10% of the detected significant markers are located within a distance of 10 kbp to the causal locus. A larger sample size may increase the power to detect such small additive effects in genome-wide scans. Despite the smaller additive effect in model (ii), the number of significant markers within 10 kbp of the QTN was comparable to model (i). Additionally, there is an increased number of significant associations further than 10 kbp from the QTNs. These may represent false positive associations caused by epistasis, such as markers that are in strong linkage disequilibrium with the fictive epistatic marker [27]. The highest power was observed in the third model. QTNs on a common haploblock with epistatic effects create a strong joint QTL and therefore in more than 75% of simulations, a significant marker was located within a distance of 10 kbp to the causal locus. The results show that single marker association methods as EMMAX are able to detect QTNs with small additive effects and a strong positive epistatic interaction. However, in certain situations larger samples than simulated sizes are needed and some results may be confounded by false positives as discussed earlier [27].

On average, a single simulation ran 4 min with GENOME[8] and 2 min with phenosim on a single core of an Intel Xeon X5650 (2.66 GHz) Processor. To compare this running time with other software tools, we simulated two QTNs and 249,998 neutral loci in a population of 500 diploid individuals using quantiNemo[12]. In six minutes, quantiNemo generated ~120 generations. As the expected coalescent time for a sample is *~ *4*N_e _*generations [28], this is by far not enough to get a realistic variation pattern comparable to what can be achieved by GENOME in the same time. Although forward simulations like quantiNemo allow more complex demographic, selection and trait scenarios, the combination of coalescent simulators and phenosim is much more suitable for generating multiple simulations of large sample sizes.

## Conclusions

Demographic effects, genetic architecture, selection, and different mutation and recombination rates affect the ability to detect the genetic basis of complex traits in natural populations [4]. Such population genetic parameters can now be estimated from genome-wide marker sets prior to further analyses. Since GWAS are widely used in plant and animal genetics, there is a great interest in assessing the power of a particular study or method. Using coalescent simulations in conjunction with phenosim, one can investigate the statistical power and other characteristics of GWAS methods efficiently. Additionally, as different causal markers may contribute different effects to a trait, the essential sample size and number of markers to detect a certain pattern can be estimated.

## Competing interests

The authors declare that they have no competing interests.

## Availability and requirements

• **Project name: **phenosim

• **Project home page: **http://evoplant.uni-hohenheim.de

• **Operating system(s): **Platform independent

• **Programming language: **Python

• **Other requirements: **Python 2.X

• **License: **no license required

• **Any restrictions to use by non-academics: **none

## Authors' contributions

TG, IG and KJS conceived the project. TG and IG designed the software. TG wrote the code. IG analyzed the data. KJS supervised the project. All authors contributed to writing of the manuscript. All authors read and approved the final manuscript.

## Supplementary Material

Additional file 1 **phenosim v0.15**. The archive includes the current version of phenosim as well as a documentation of its usage. For updated versions, please visit the authors' website http://evoplant.uni-hohenheim.de. Click here for file

## References

[B1] Hindorff La Sethupathy P Junkins Ha Ramos EM Mehta JP Collins FS Manolio TA Potential etiologic and functional implications of genome-wide association loci for human diseases and traits Proceedings of the National Academy of Sciences of the United States of America 2009 106 23 9362 7 10.1073/pnas.0903103106 19474294 PMC2687147

[B2] Atwell S Huang YS Vilhjálmsson BJ Willems G Horton M Li Y Meng D Platt A Tarone AM Hu TT Jiang R Muliyati NW Zhang X Amer MA Baxter I Brachi B Chory J Dean C Debieu M de Meaux J Ecker JR Faure N Kniskern JM Jones JDG Michael T Nemri A Roux F Salt DE Tang C Todesco M Traw MB Weigel D Marjoram P Borevitz JO Bergelson J Nordborg M Genome-wide association study of 107 phenotypes in *Arabidopsis thaliana inbred lines* Nature 2010 465 7298 627 31 10.1038/nature08800 20336072 PMC3023908

[B3] Stranger BE Stahl Ea Raj T Progress and Promise of Genome-wide Association Studies for Human Complex Trait Genetics Genetics 2010 187 2 367 383 21115973 10.1534/genetics.110.120907 PMC3030483

[B4] Wang WYS Barratt BJ Clayton DG Todd JA Genome-wide association studies: theoretical and practical concerns Nature reviews Genetics 2005 6 2 109 18 10.1038/nrg1522 15716907

[B5] Hudson RR Generating samples under a Wright-Fisher neutral model of genetic variation Bioinformatics 2002 18 337 338 10.1093/bioinformatics/18.2.337 11847089

[B6] Hellenthal G Stephens M msHOT: modifying Hudson's ms simulator to incorporate crossover and gene conversion hotspots Bioinformatics 2007 23 4 520 1 10.1093/bioinformatics/btl622 17150995

[B7] Ewing G Hermisson J MSMS: A Coalescent simulation program including recombination, demographic structure, and selection at a single locus Bioinformatics 2010 26 16 2064 2065 10.1093/bioinformatics/btq322 20591904 PMC2916717

[B8] Liang L Zöllner S Abecasis GR GENOME: a rapid coalescent-based whole genome simulator Bioinformatics 2007 23 12 1565 7 10.1093/bioinformatics/btm138 17459963

[B9] Mailund T Schierup MH Pedersen CNS Mechlenborg PJM Madsen JN Schauser L CoaSim: A flexible environment for simulating genetic data under coalescent models BMC Bioinformatics 2005 6 252 10.1186/1471-2105-6-252 16225674 PMC1274299

[B10] Chadeau-Hyam M Hoggart CJ O'Reilly PF Whittaker JC Iorio MD Balding DJ Fregene: simulation of realistic sequence-level data in populations and ascertained samples BMC Bioinformatics 2008 9 364 10.1186/1471-2105-9-364 18778480 PMC2542380

[B11] Lambert BW Terwilliger JD Weiss KM ForSim: a tool for exploring the genetic architecture of complex traits with controlled truth Bioinformatics 2008 24 16 1821 2 10.1093/bioinformatics/btn317 18565989 PMC2732213

[B12] Neuenschwander S Hospital F Guillaume F Goudet J quantiNemo: an individual-based program to simulate quantitative traits with explicit genetic architecture in a dynamic metapopulation Bioinformatics 2008 24 13 1552 3 10.1093/bioinformatics/btn219 18450810

[B13] Peng B Amos CI Forward-time simulation of realistic samples for genome-wide association studies BMC Bioinformatics 2010 11 442 10.1186/1471-2105-11-442 20809983 PMC2939614

[B14] van Rossum G Python Reference manual 1995 Amsterdam: CWI (Centre for Mathematics and Computer Science)

[B15] Nordborg M Tavaré S Linkage disequilibrium: what history has to tell us Trends in Genetics 2002 18 2 83 90 10.1016/S0168-9525(02)02557-X 11818140

[B16] Besenbacher S Mailund T Schierup MH Local phylogeny mapping of quantitative traits: higher accuracy and better ranking than single-marker association in genomewide scans Genetics 2009 181 2 747 53 19064712 10.1534/genetics.108.092643 PMC2644962

[B17] Hudson RR Kaplan NL Statistical properties of the number of recombination events in the history of a sample of DNA sequences Genetics 1985 111 147 64 4029609 10.1093/genetics/111.1.147 PMC1202594

[B18] Mailund T Besenbacher S Schierup MH Whole genome association mapping by incompatibilities and local perfect phylogenies BMC Bioinformatics 2006 7 454 10.1186/1471-2105-7-454 17042942 PMC1624851

[B19] Kang HM Zaitlen Na Wade CM Kirby A Heckerman D Daly MJ Eskin E Efficient control of population structure in model organism association mapping Genetics 2008 178 3 1709 23 10.1534/genetics.107.080101 18385116 PMC2278096

[B20] Kang HM Sul JH Service SK Zaitlen NA Kong SY Freimer NB Sabatti C Eskin E Variance component model to account for sample structure in genome-wide association studies Nature Genetics 2010 42 4 348 354 10.1038/ng.548 20208533 PMC3092069

[B21] Purcell S Neale B Toddbrown K Thomas L Ferreira M Bender D Maller J Sklar P Debakker P Daly M PLINK: A tool set for whole-genome association and population-based linkage analyses The American Journal of Human Genetics 2007 81 3 559 575 10.1086/519795 17701901 PMC1950838

[B22] Abecasis GR Cardon LR Cookson WO A general test of association for quantitative traits in nuclear families American journal of human genetics 2000 66 279 92 10.1086/302698 10631157 PMC1288332

[B23] Abecasis GR Cherny SS Cookson WO Cardon LR Merlin-rapid analysis of dense genetic maps using sparse gene flow trees Nature Genetics 2002 30 97 101 10.1038/ng786 11731797

[B24] Bradbury PJ Zhang Z Kroon DE Casstevens TM Ramdoss Y Buckler ES TASSEL: software for association mapping of complex traits in diverse samples Bioinformatics 2007 23 19 2633 5 10.1093/bioinformatics/btm308 17586829

[B25] Kim S Plagnol V Hu TT Toomajian C Clark RM Ossowski S Ecker JR Weigel D Nordborg M Recombination and linkage disequilibrium in *Arabidopsis thaliana* Nature Genetics 2007 39 9 1151 5 10.1038/ng2115 17676040

[B26] Li Y Huang Y Bergelson J Nordborg M Borevitz JO Association mapping of local climate-sensitive quantitative trait loci in *Arabidopsis thaliana* Proceedings of the National Academy of Sciences 2010 107 49 21199 21204 10.1073/pnas.1007431107 PMC3000268 21078970

[B27] Platt A Vilhjálmsson BJ Nordborg M Conditions under which genome-wide association studies will be positively misleading Genetics 2010 186 3 1045 1052 10.1534/genetics.110.121665 20813880 PMC2975277

[B28] Nordborg M D. J. Balding, M. J. Bishop, and C. Cannings (Editors), Handbook of Statistical Genetics Coalescent theory 2001 New York: John Wiley and Sons 179 212

