# Supplementary material for: phenosim - A software to simulate phenotypes for testing in genome-wide association studies
Source: BMC Bioinformatics. 2011 Jun 29;12:265. doi: 10.1186/1471-2105-12-265 (PMC3150295; doi:10.1186/1471-2105-12-265)
Supplement: Additional file 1 — phenosim v0.15. The archive includes the current version of phenosim as well as a documentation of its usage. For updated versions, please visit the authors' website http://evoplant.uni-hohenheim.de. [file 1471-2105-12-265-S1.ZIP › phenosim_doc.pdf]

# phenosim
*Documentation*

June 20, 2011

# 1 Introduction

This document describes the use of the software `phenosim`, a tool to add phenotypes to simulated genotypes. phenosim reads the output of coalescent simulators and simulates a qualitative or quantitative phenotype according to user-defined parameters. Finally, the output of phenosim is written in file formats for various widely used association softwares.

## 1.1 Disclaimer

This software is open-source, so feel free to look at the source code and modify it. The code is not written by professional programmers and therefore it neither looks nor behaves like professional code. However, it should provide the desired functionality.

We hope that the software is useful, but we provide it without any warranty for correctness, stability or anything else.

Please report bugs to `torsten.guenther@uni-hohenheim.de`

## 1.2 Setup

The software was coded in Python 2.6 and uses some of Python's standard libraries. It was tested on a Linux environment, but since Python is portable, it should run on other OS as well.

As you read this document, you already downloaded the package from `http://evoplant.uni-hohenheim.de`. `cd` to your download directory, unpack the archive and make the file phenosim.py executable (`chmod +x phenosim.py`). Then typing

```
./phenosim.py
```

will print a general description of `phenosim`'s options and you will see, that you unpacked the archive correctly. Then you are ready to use `phenosim` in its directory.

In order to make `phenosim` available across your entire system type `export PATH=$PATH:$PWD`. If you want to make this setting permanent, go to your home-directory and add the line `export PATH=$PATH:<path to phenosim>` (insert the directory of phenosim) to your .bashrc file.

### 1.2.1 Windows

Most of the settings described above apply only for Unix-like systems (Unix, Linux, MacOS etc.) and not for Windows. Windows users first have to make sure that Python (van Rossum, 1995) is installed on their computer. Python can be downloaded from `http://www.python.org/`. After unpacking the archive, Windows users need to make `phenosim.py` executable: right-click on the file, select 'Properties' and then uncheck the box 'Read-only'. In order to make `phenosim` available across your entire system, you have to add `phenosim`'s directory to your system's PATH. Please consult your system administrator or Google for changing the PATH in your particular windows version.

### 1.3 Citation

When using `phenosim`, please cite the following paper:

T. Günther, I. Gawenda and K. J. Schmid: `phenosim` - A software to simulate phenotypes for testing in genome-wide association studies. *submitted*

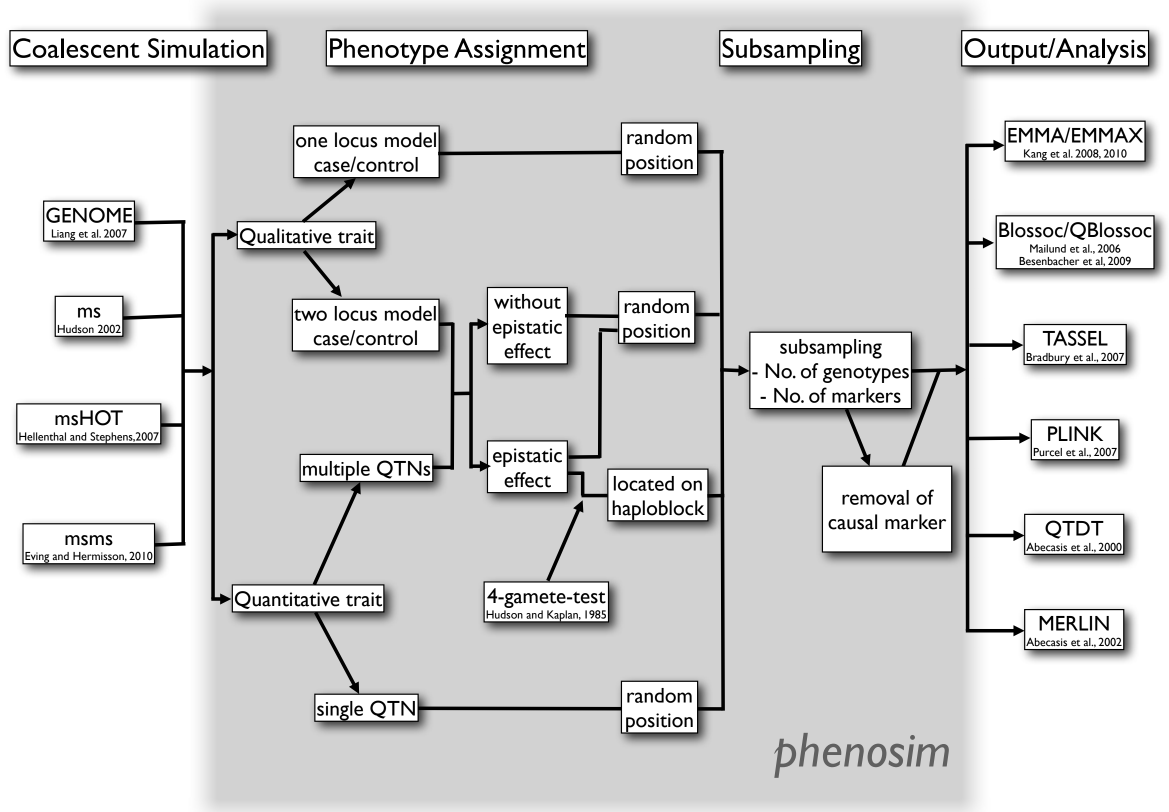


Figure 1: Flowchart of the `phenosim` pipeline

## 2 Files

### 2.1 Input

`phenosim` reads an input file defined using the `-f` option. This file can be the output of *ms* (Hudson, 2002) ( `-i M` ), *msHOT* (Hellenthal and Stephens, 2007) ( `-i M` ), *msms* (Ewing and Hermisson, 2010) ( `-i M` ) or GENOME (Liang et al, 2007) ( `-i G` ). Files containing multiple simulation runs may be used as well as files containing just a single run. Under the default setting, the individuals are treated as haploids, by setting `-d 1` they are treated as diploids. Diploid individuals are created by combining two simulated chromosomes to a joint genotype.

### 2.2 Output

`phenosim` writes the genotypes and phenotypes in file formats compatible for EMMA (Kang et al, 2008) (`-o E`), BLOSSOC (Mailund et al, 2006) (`-o B`), TASSEL 3.0 (Bradbury et al, 2007) (`-o T`), QTDT (Abecasis et al, 2000), MERLIN (Abecasis et al, 2002), EMMAX (Kang et al, 2010) and PLINK (Purcell et al, 2007) (`-o P`). The prefix of the output files can be defined by the user (`--outfile`). The file names consist of the name of the prefix, the simulation number and their particular suffix. Additionally, a file

.causal is created which includes the position, index, minor allele frequency (MAF) and the effect of the causal marker(s).

| **Program** | **Option** | **File suffixes** | **Comment** |
|---|---|---|---|
| EMMA | -o E | .emma_geno, .emma_pheno | missing data coded as 'NA', no diploids supproted! |
| BLOSSOC | -o B | .blossoc_pos, .blossoc_geno | no missing data or diploids supported! |
| PLINK/EMMAX | -o P | .ped, .map, .pheno | case/control-status coded as 0/1, use the –1 flag in PLINK; .pheno is needed for EMMAX (Kang et al, 2010); missing alleles coded as '0' |
| QTDT/MERLIN | -o Q | .ped, .map, .dat | case/control-status coded as 0/1 missing alleles coded as '0' |
| TASSEL | -o T | .trait, .poly | file formats for TASSEL 3.0; missing alleles coded as '?' |

# 3 Simulation of Phenotypes

## 3.1 Qualitative traits

Qualtitative traits are analyzed in case/control studies.

### 3.1.1 One-locus model

`phenosim` offers the possibility to simulate a single causal marker for a qualitative trait (`-q 0`) and to define two different probabilities $P(\text{affected}|\text{wildtype})$ (`--prob_wt`) and $P(\text{affected}|\text{(homozygous) mutant})$ (`--prob_mut`), if diploids are simulated, a third probability $P(\text{affected}|\text{heterozygous})$ (`--prob_het`) is requested.

**Example:** `phenosim.py -f out.txt -i M -o P -q 0 -d 1 --prob_wt 0.1 --prob_het 0.3 --prob_mut 0.5`
This example reads a file out.txt from *ms* and simulates diploid individuals with a qualitative phenotype with $P(\text{affected}|\text{wildtype}) = 0.1$, $P(\text{affected}|\text{heterozygous}) = 0.3$ and $P(\text{affected}|\text{mutant}) = 0.5$ and writes the output in PLINK compatible files.

### 3.1.2 Two-locus model

Models including two risk loci are supported by `phenosim` as well. For such models, the probabilities of being affected are described in a penetrance table for each possible allelic combination. The tables have the format 2x2 for haploid and 3x3 for diploid indivduals. The structure of such table for diploids is given in the table below, where 0 corresponds to a homozygous ancestral, 1 corresponds to heterozygous and 2 corresponds to homozygous derived genotypes.

| genotype | 0 | 1 | 2 |
|---|---|---|---|
| 0 | $p_{00}$ | $p_{10}$ | $p_{20}$ |
| 1 | $p_{01}$ | $p_{11}$ | $p_{21}$ |
| 2 | $p_{02}$ | $p_{12}$ | $p_{22}$ |

The table is read from a file (`--pene_tab`) which includes the penetrances separated by spaces or tabulators. An example for such file is given in *domdom_penetrance.txt*, which corresponds to a dominant-dominant model for diploids. The two risk loci may also be located on a common haplotype (`--epi_hapl 1`, see section 3.2.3 for details).

**Example:** `phenosim.py -f out.txt -i M -o Q -q 0 -d 1 -n 2 --pene_tab domdom_penetrance.txt`
This example reads a file out.txt from *ms* and simulates diploid individuals with a qualitative phenotype with penetrances defined in `domdom_penetrance.txt` and writes the output in QTDT compatible files.

### 3.1.3 Number of cases

Real case/control studies are usually set up by selecting a particular number of cases and controls from a population without knowledge about their genotype. The disease loci, however, are frequently caracterized by a low allele frequency of the risk allele. Therefore, restricting the allele frequency of the risk locus to a low frequency range (see section 3.2.1 for an example) may result in a very low number of cases. Selecting higher frequency ranges would bias the genealogy of the risk locus, so we suggest a different workaround.

The user should start by simulating a larger sample using the coalescent package of choice. As a rule of thumb, this sample should have a minimum size of $\frac{\text{number of cases} \times \text{penetrance of risk genotype}}{\text{risk allele frequency}}$. When applying `phenosim` to this simulated population, the user can then use `--min_cases` to determine the desired minimum number of cases and the use `--n_gt` (see section 4) to restrict the final sample to the favoured size. When `--min_cases` is used, the minimum number of cases is sampled directly after calculation of the affection status for all indivduals and the remaining number of indivduals is randomly chosen from the entire population. If less cases than `--min_cases` were created, an error message is giving which suggests to either simulate a larger population or change the risk locus' frequency range.

**Example:** `phenosim.py -f out.txt -i M -o P -q 0 -d 1 --prob_wt 0.1 --prob_het 0.3 --prob_mut 0.5 --maf_r 0.05,0.1 --n_gt 250 --min_cases 100`
This example reads a file out.txt from *ms* and simulates diploid individuals with a qualitative phenotype with $P(\text{affected}|\text{wildtype}) = 0.1$, $P(\text{affected}|\text{heterozygous}) = 0.3$ and $P(\text{affected}|\text{mutant}) = 0.5$ caused by a risk locus with a minor allele frequency between 5 and 10 percent, subsamples 250 individuals of which at least 100 are cases and writes the output in PLINK compatible files.

## 3.2 Quantitative traits

The simulation of quantitative traits (`-q 1`) follows a generalized version of the formulas introduced by Besenbacher et al (2009) for additive effects between mutiple quantitative trait nucleotides (QTNs).

### 3.2.1 Haploid individuals

The indivduals are treated as haploids. The $j$-th QTN explains a variance proportion of $\pi_j$ and has a derived allele frequency of $f_j$. If the allelic state of the $i$-th indivdual at the $j$-th QTN is then $a_{ij}$, the phenotype of indivdual $i$ is calculated as:

$$Y_i = \sqrt{1 - \sum_j \pi_j} \cdot N(0,1) + \sum_j a_{ij} \sqrt{\frac{\pi_j}{f_j(1-f_j)}}$$

**Example:** `phenosim.py -f out.txt -i M -o B -q 1 -n 2 -v 0.2,0.2 --maf_r 0.2,0.5`
This example reads a file out.txt from *ms* and simulates a quantitative phenotype with two QTNs, both with an effect of 20% and with a minor allele frequency(MAF) between 0.2 and 0.5. The output is written in Blossoc compatible files.

**Attention!** For the options `-v` and `--maf_r` please only use commas to separate the values. Use no spaces!

### 3.2.2 Diploid indivduals

To create diploid individuals, `-d 1` has to be set. The diploids are created by combining two simualted chromosomes to a common genotype. The $j$-th QTN explains a variance proportion of $\pi_j$ and has a derived allele frequency of $f_j$. The allelic state of the $i$-th indivdual at the $j$-th QTN is then defined as follows

$$a_{ij} := \begin{cases} 1, & \text{if homozygous 1,} \\ 0, & \text{if heterozygous,} \\ -1 & \text{if homozygous 0.} \end{cases}$$

The phenotype of indivdual $i$ is calculated as:

$$Y_i = \sqrt{1 - \sum_j \pi_j} \cdot N(0,1) + \sum_j a_{ij} \sqrt{\frac{\pi_j}{2 \cdot f_j(1-f_j)}}$$

**Example:** `phenosim.py -f out.txt -i M -o Q -q 1 -d 1 -n 2 -v 0.2,0.2 --maf_r 0.2,0.5`
This example reads a file out.txt from *ms* and simulates diploid indivduals with a quantitative phenotype with two QTNs, both with an effect of 20% and with a minor allele frequency(MAF) between 0.2 and 0.5. The output is written in QTDT compatible files.

**Attention!** For the options `-v` and `--maf_r` please only use commas to separate the values. Use no spaces!

### 3.2.3 Epistasis

`phenosim` offers the option to simulate epistatic effects between two QTNs (`-e 1`). The epistatic effect is modelled as a fictive third QTN where the causal allele is only present if both basal QTNs carry the derived state on at least one chromosome. The epistatic effect (`--epi_eff 0.X`) is additive. Additionally, one can choose that both QTNs are located on a common haploblock defined by the four-gamete test (`--epi_hapl 1`), which could be the case if there is allelic heterogenity of two QTNs within one gene.

**Example:** `phenosim.py -f out.txt -i G -o P -q 1 -n 2 -v 0.1,0.1 -e 1 --epi_eff 0.3 --epi_hapl 1`
This example reads a file out.txt from GENOME and simulates a quantitative phenotype with two QTNs, both with an effect of 10%. These QTNs are located on a common haploblock and have an epistatic effect of 30%. The output is written in PLINK compatible files.

**Modifying the code for epistasis** `phenosim` models the epistatic effect as an additional QTN whose allelic state is defined by a logical function based on the two basal QTNs. In the default implementation, this function is defined as a logical *AND* between the alleles. Other logical operations may be implemented by modifying the code as follows.

The respective code is located in the lines 202 to 210 in the file `phenotyper.py`:

```
...
if diploid:
     if alleles[0]>0 and alleles[1]>0:
          root=epi_effect/(epi_freq*(1-epi_freq))
          phen+=1*math.sqrt(root)
     else:
          if alleles[0] and alleles[1]:
...
```

To modify the logical operation, just modify the lines containing `and`. The `and` may be replaced by an `or` and `not` may be written before the variables to obtain their negation.

# 4 Sampling

In addition to the simulation of phenotypes, `phenosim` can be used to subsample a certain number of markers (`--n_m X`) and genotypes (`--n_gt Y`) from the simulated genotypes. Additionally, the sampled markers may be selected according to a minimum MAF (`--maf_c Z`) and a certain proportion of alleles may be missing (`--miss X`). To ensure that the causal marker(s) are removed from the sample, `-r 1` has to be set. This is fairly realistic, since it is very unlikely that the causal mutation itself is genotyped in a genome-wide study.

**Example:** `phenosim.py -f out.txt -i G -o P -q 1 -n 2 -v 0.1,0.1 --maf_c 0.05 --n_m 1000 --n_gt 100 -r 1`
This example reads a file out.txt from GENOME and simulates a quantitative phenotype with two QTNs,

both with an effect of 10%. 1000 markers with an minor allele frequency $\geq 0.05$ and 100 genotypes are then subsampled and the two causal markers are removed from this sample. The output is written in PLINK compatible files.

## 5 Summary of options

| Option | Description |
|---|---|
| **general** | |
| `-f` | name of input file |
| `-i` | type of input file (”G” for GENOME, ”M” for *ms*, *msHOT* and *msms*) (default: M) |
| `-o` | type of output file (”B” for Blossoc, ”E” for EMMA, ”P” for PLINK/EMMAX, ”T” Tassel 3.0, “Q“ for QTDT/MERLIN; comma separated list of these letters is also supported) (default: E) |
| `-d` | logical value for ploidy, indivduals are either haploid (0) or diploid (1) (default: 0) |
| `--outfile` | prefix for the output files (default: name of input file) |
| `-q` | logical value if quantitaive (1) or qualitative (0) phenotypes should be simulated (default: 1) |
| `--causal_pos` | predefined position of causal marker, then the marker closest to this position is selected as causal marker (only possible for a single causal marker) (no default) |
| | |
| **qualitative traits** | |
| `--prob_wt` | (if q=0) probability of the wild type being affected (no default) |
| `--prob_mut` | (if q=0) probability of the mutant being affected (no default) |
| `--prob_het` | (if q=0 and d=1) probability of the heterozygous being affected (no default) |
| `--pene_tab` | (if q=0 and n=2) filename of penetrance table (no default) |
| `--min_cases` | (if q=0) minimum number of cases in the final sample (no default) |
| | |
| **quantitative traits** | |
| `-n` | (if q=1) number of simulated QTNs (default: 1) |
| `-v` | (if q=1) proportion of variance explained by the QTNs (if multiple QTNs are simulated, separate the values by commas, use no spaces) (default: 0.5) |
| `--dominant` | (if q=1) additive (0) or dominant (1) model used (default: 0) |
| | |
| **epistasis** | |
| `-e` | (if q=1 and numqtn=2) logical value if epistatic interaction between the two QTNs is wanted (1) or not (0) (default: 0) |
| `--epi_eff` | (if q=1 and n=2) proportion of variance explained by the epistatic effect (default: 0) |
| | |
| `--epi_hapl` | (and n=2) logical value indicating if the epistatic causal markers should lie on a common haploblock (1) or not (0) (default: 0) |
| | |
| **sampling** | |
| `--maf_r` | MAF range for causal markers (upper and lower bound, separated by a comma, no space) (default: 0.05,1.0) |
| `--maf_c` | minimum MAF for sampled markers (default: 0.05) |
| `--n_m` | number of sampled markers (default: all simulated markers) |
| `--n_gt` | number of sampled genotypes (default: all simulated genotypes) |
| `--miss` | proportion of missing alleles (default: 0.0) |
| `-r` | logical value if causal markers should be removed from the sample (1) or not (0) (default: 0) |

## References


Abecasis GR, Cardon LR, Cookson WO (2000) A general test of association for quantitative traits in nuclear families. American journal of human genetics 66(1):279–92, DOI 10.1086/302698

Abecasis GR, Cherny SS, Cookson WO, Cardon LR (2002) Merlin–rapid analysis of dense genetic maps using sparse gene flow trees. Nature Genetics 30(1):97–101, DOI 10.1038/ng786

Besenbacher S, Mailund T, Schierup MH (2009) Local phylogeny mapping of quantitative traits: higher accuracy and better ranking than single-marker association in genomewide scans. Genetics 181(2):747–53, DOI 10.1534/genetics.108.092643

Bradbury PJ, Zhang Z, Kroon DE, Casstevens TM, Ramdoss Y, Buckler ES (2007) TASSEL: software for association mapping of complex traits in diverse samples. Bioinformatics 23(19):2633–5, DOI 10.1093/bioinformatics/btm308

Ewing G, Hermisson J (2010) MSMS: A Coalescent simulation program including recombination, demographic structure, and selection at a single locus. Bioinformatics 26(16):2064–2065, DOI 10.1093/bioinformatics/btq322

Hellenthal G, Stephens M (2007) msHOT: modifying Hudson's ms simulator to incorporate crossover and gene conversion hotspots. Bioinformatics 23(4):520–1, DOI 10.1093/bioinformatics/btl622

Hudson RR (2002) Generating samples under a Wright-Fisher neutral model of genetic variation. Bioinformatics 18:337–338

Kang HM, Zaitlen Na, Wade CM, Kirby A, Heckerman D, Daly MJ, Eskin E (2008) Efficient control of population structure in model organism association mapping. Genetics 178(3):1709–23, DOI 10.1534/genetics.107.080101

Kang HM, Sul JH, Service SK, Zaitlen NA, Kong SY, Freimer NB, Sabatti C, Eskin E (2010) Variance component model to account for sample structure in genome-wide association studies. Nature Genetics (March), DOI 10.1038/ng.548

Liang L, Zöllner S, Abecasis GR (2007) GENOME: a rapid coalescent-based whole genome simulator. Bioinformatics 23(12):1565–7, DOI 10.1093/bioinformatics/btm138

Mailund T, Besenbacher S, Schierup MH (2006) Whole genome association mapping by incompatibilities and local perfect phylogenies. BMC Bioinformatics 7:454, DOI 10.1186/1471-2105-7-454

Purcell S, Neale B, Toddbrown K, Thomas L, Ferreira M, Bender D, Maller J, Sklar P, Debakker P, Daly M (2007) PLINK: A tool set for whole-genome association and population-based linkage analyses. The American Journal of Human Genetics 81(3):559–575, DOI 10.1086/519795

van Rossum G (1995) Python Reference manual. CWI (Centre for Mathematics and Computer Science), Amsterdam
